# ASC-dependent inflammasomes contribute to immunopathology and mortality in herpes simplex encephalitis

**DOI:** 10.1371/journal.ppat.1009285

**Published:** 2021-02-01

**Authors:** Cooper K. Hayes, Douglas R. Wilcox, Yuchen Yang, Grace K. Coleman, Melissa A. Brown, Richard Longnecker

**Affiliations:** 1 Department of Microbiology and Immunology, Northwestern University Feinberg School of Medicine, Chicago, IL, United States of America; 2 Department of Neurology, Brigham and Women’s Hospital, Boston, MA, United States of America; 3 Department of Neurology, Massachusetts General Hospital, Boston, MA, United States of America; 4 Department of Neurology, Harvard Medical School, Boston, MA, United States of America; University of North Carolina at Chapel Hill, UNITED STATES

## Abstract

Herpes simplex virus encephalitis (HSE) is the most common cause of sporadic viral encephalitis, and despite targeted antiviral therapy, outcomes remain poor. Although the innate immune system is critical for restricting herpes simplex virus type I (HSV-1) in the brain, there is evidence that prolonged neuroinflammation contributes to HSE pathogenesis. In this study, we investigated the contribution of inflammasomes to disease pathogenesis in a murine model of HSE. Inflammasomes are signaling platforms that activate the pro-inflammatory cytokines interleukin-1β (IL-1β) and IL-18. We found that mice deficient in the inflammasome adaptor protein, apoptosis-associated speck-like protein containing a caspase activation and recruitment domain (ASC), had significantly improved survival and lower levels of IL-1β and IL-18 in the brain. Importantly, this difference in survival was independent of viral replication in the central nervous system (CNS). We found that microglia, the resident macrophages of the CNS, are the primary mediators of the ASC-dependent inflammasome response during infection. Using in vitro glial infections and a murine HSE model, we demonstrate that inflammasome activation contributes to the expression of chemokine (C-C motif) ligand 6 (CCL6), a leukocyte chemoattractant. The lower concentration of CCL6 in the brains of ASC^-/-^ mice correlated with lower numbers of infiltrating macrophages during infection. Together, these data suggest that inflammasomes contribute to pathogenic inflammation in HSE and provide a mechanistic link between glial inflammasome activation and leukocyte infiltration. The contribution of inflammasomes to survival was independent of viral replication in our study, suggesting a promising new target in combating harmful inflammation in HSE.

## Author summary

Although the immune system is critical for controlling herpes simplex virus (HSV) infection in the central nervous system (CNS), a prolonged or excessive immune response may be harmful. We studied the contribution of inflammasomes, innate immune proteins that initiate a pro-inflammatory response, to detrimental neuroinflammation in a murine model of herpes simplex encephalitis (HSE). We found that inflammasomes that rely on the adaptor protein ASC contribute to mortality and neuroinflammation independent of controlling viral replication. Additionally, we demonstrate that the inflammasome response to HSV is primarily mediated by microglia and leads to an influx of macrophages in the CNS. These results provide insights into the mechanisms that drive pathogenic inflammation in HSE and suggest a promising therapeutic target in treating HSE.

## Introduction

Herpes simplex virus type I (HSV-1) is a neurotropic virus and the most common cause of viral encephalitis [1]. Despite antiviral therapy targeted to control viral replication, morbidity and mortality in herpes simplex encephalitis (HSE) remains high [2]. Although the innate immune system is critical for controlling early viral replication [3,4], there is evidence to suggest that excessive neuroinflammation may play a detrimental role during infection. In particular, the prolonged activation of microglia, the resident macrophages in the central nervous system (CNS), and recruitment of infiltrating leukocytes to the brain contribute to pathology in HSE [5,6]. Cytokines and other markers of inflammation are elevated in the cerebrospinal fluid of patients, even months after symptoms have resolved [7]. Taken together, these data suggest that the immune response to HSV in the brain represents a careful balance between controlling viral replication and immune-mediated damage [8], but the specific innate signaling pathways that contribute to this balance are unknown.

Inflammasomes are pro-inflammatory signaling platforms that respond to pathogens and host cell danger signals. Inflammasome activation is initiated by engagement of pattern-recognition receptors (PRRs) and results in the downstream activation of caspase-1, leading to the processing and release of the pro-inflammatory cytokines interleukin-1β (IL-1β) and IL-18, and a lytic form of cell death called pyroptosis [9]. Canonical inflammasomes, including the NOD-, LRR- and pyrin domain-containing 3 (NLRP3) and absent in melanoma 2 (AIM2) inflammasomes, require the adaptor protein apoptosis-associated speck-like protein containing a caspase activation and recruitment domain (ASC) [9]. The inflammasome signaling pathway is implicated in the pathogenesis of many neurologic diseases, including autoimmune encephalomyelitis, ischemic stroke, and neurodegenerative diseases [10–12].

The role of inflammasomes in the pathogenesis of viral infections is complicated, with inflammasomes shown to be protective in some disease models and detrimental in others [13–17]. Recent work has demonstrated that HSV-1 activates several inflammasomes in vitro and in vivo, including NLRP3 and AIM2 [18–22]. However, these studies have been largely limited to models of intraperitoneal and ocular infection. Intraperitoneal inoculation with high doses of HSV-1 led to higher viral titers in the brains of NLRP3^-/-^ mice, suggesting that the NLRP3 inflammasome may limit neuroinvasiveness [22]. In a model of the ocular herpes stromal keratitis, NLRP3^-/-^ mice demonstrated a more robust early immune response, suggesting that NLRP3 plays an immunomodulatory role in corneal infection [23]. The role that inflammasomes play in HSV infection of the central nervous system, and the specific cell populations that drive this response, are unknown.

In this study, we investigated the contribution of inflammasomes in a murine model of HSE. We found improved survival in ASC^-/-^ mice compared to wild-type (WT), but no difference in survival between NLRP3^-/-^ and AIM2^-/-^ mice. These results corresponded with a decrease in pro-inflammatory cytokines in the brains of ASC^-/-^ mice. Interestingly, this difference in immune response was independent of viral replication. We identified microglia as key mediators of the inflammasome response during HSE and found that glial inflammasome activation is necessary for the expression of the monocyte chemoattractant CCL6. Accordingly, ASC^-/-^ mice had reduced numbers of infiltrating macrophages during HSE.

## Results

### The inflammasome adaptor protein ASC contributes to mortality during HSE

**Fig 1:**
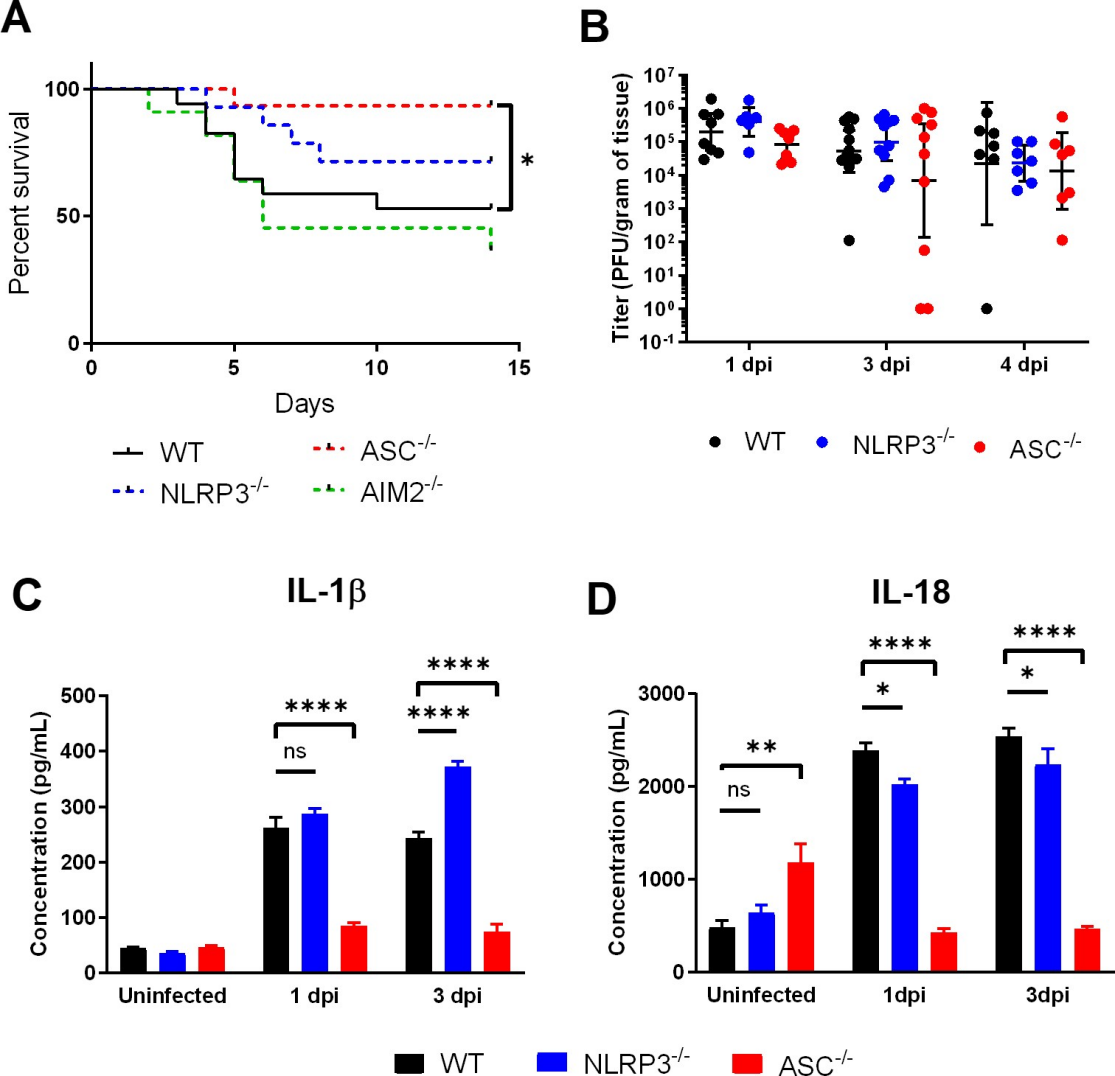
ASC-dependent inflammasomes contribute to the pathogenesis of HSV-1 encephalitis. (A) Survival curve of WT (n = 17), NLRP3^-/-^ (n = 14), AIM2^-/-^ (n = 11), and ASC^-/-^ (n = 15) mice infected intracranially with 3x10^4^ PFU HSV-1 KOS. Results are combined from seven inoculations. (B) Whole brain titers of infected WT, NLRP3^-/-^, and ASC^-/-^ mice at days 1, 3, and 4 post-infection (n = 7–12 per group, 8 inoculations). (C) Whole brain IL-1β and (D) IL-18 from WT, NLRP3^-/-^, and ASC^-/-^ mice either before infection or at days 1 and 3 post-infection (n = 4–5 per group). Values are expressed as means ± SEM (**P < 0.01, ***P < 0.001, ****P < 0.0001).

The role of inflammasomes in the pathogenesis of viral infections depends on both the virus and the target organ host response, with inflammasomes serving a protective role in some disease models and a detrimental one in others [14,17,24]. IL-1β knockout mice exhibit increased susceptibility to infection in an intranasal model of HSE [25]. Therefore, we hypothesized that inflammasome knockout mice would also be more susceptible to disease in a model of herpes simplex encephalitis. Direct intracranial inoculation of HSV-1 in a murine model of HSE was used to prevent any confounding impact of peripheral replication on neuroinvasiveness. WT, NLRP3^-/-^, AIM2^-/-^, and ASC^-/-^ mice were infected and monitored for survival over 14 days. WT mice began to succumb to disease on day 4 post-infection with a mean survival time of 9.9 days, and 52.9% survived to the experimental endpoint (Fig 1A). Notably, ASC^-/-^ mice had significantly improved survival compared to WT, with 93.3% of ASC^-/-^ mice surviving until the experimental endpoint and a mean survival time of 13.4 days. NLRP3^-/-^ and AIM2^-/-^ mice did not have statistically significant differences in survival compared to WT mice (71.4% and 36.4%, respectively). This suggests that although the individual inflammasome sensor components NLRP3 and AIM2 are dispensable for HSE survival, the common inflammasome adaptor ASC contributes to mortality.

A possible explanation for the improved survival in ASC^-/-^ mice is that ASC-dependent inflammasomes directly affect HSV-1 replication in the CNS. To test this, we infected WT, NLRP3^-/-^, and ASC^-/-^ mice and determined viral replication in brain tissue at various time points until average time to mortality. No difference in viral titer was found at 1, 3, or 4 days post-infection, indicating that neither NLRP3 nor ASC contribute to controlling HSV-1 replication in the CNS (Fig 1B). These data suggest that differences in mortality between WT and ASC^-/-^ mice are due to the immunomodulatory function of ASC, rather than by affecting viral replication.

Following inflammasome-mediated activation of caspase-1, pro-IL-1β and IL-18 are cleaved to their activated forms to serve as the downstream mediators of the inflammasome signaling pathway. To determine the contribution of ASC-dependent inflammasomes to IL-1β and IL-18 production in HSE, we assayed mice early after infection and prior to mortality (days 1 and 3). As expected, IL-1β was low in the brains of uninfected mice but rose sharply in WT mice at days 1 and 3 post-infection (Fig 1C). ASC^-/-^ mice had significantly lower IL-1β levels than WT at both time points, suggesting that ASC-dependent inflammasomes are essential for IL-1β production during HSE. NLRP3^-/-^ mice expressed higher levels of IL-1β than WT at day 3 post-infection. This result suggests that NLRP3 does not contribute to IL-1β production in the brain, and may actually play an immunomodulatory role to regulate IL-1β production, as previously shown in ocular HSV-1 infection [23]. IL-18 concentrations followed a similar pattern, although ASC^-/-^ mice had significantly higher levels than WT at the uninfected baseline (Fig 1D). At days 1 and 3 post-infection, WT mice had significantly higher levels of brain IL-18 than NLRP3^-/-^ and ASC^-/-^ mice, demonstrating that ASC-dependent inflammasomes are also essential for IL-18 production during HSE.

### Microglial ASC-dependent inflammasomes are activated during HSE

We next sought to determine which cell types in the CNS mediate the ASC-dependent inflammasome response during HSE. Microglia are resident macrophages of the CNS and are known to secrete IL-1β under pro-inflammatory conditions [26]. Additionally, microglia have been identified as key contributors to the inflammasome response in a model of traumatic brain injury [27]. Thus, we hypothesized that microglia and infiltrating leukocytes would be the primary cell types with active inflammasome complexes during HSE. To test this hypothesis, we utilized a fluorescently-labeled inhibitor of caspase activity (FLICA) assay combined with traditional flow cytometric analysis to label cells with active caspase-1. The FAM-FLICA reagent is a membrane-permeable probe that irreversibly binds to activated caspase, selectively labeling cells with activated inflammasomes. At day 3 post-infection, single-cell suspensions of dissociated brain cells were labeled with the FAM-FLICA reagent and fluorescent antibodies to identify resident CD45^mid^ CD11b^+^ microglia and CD45^lo^ ACSA-2^+^ astrocytes, as well as infiltrating CD45^hi^ CD11b^+^ leukocytes.

**Fig 2:**
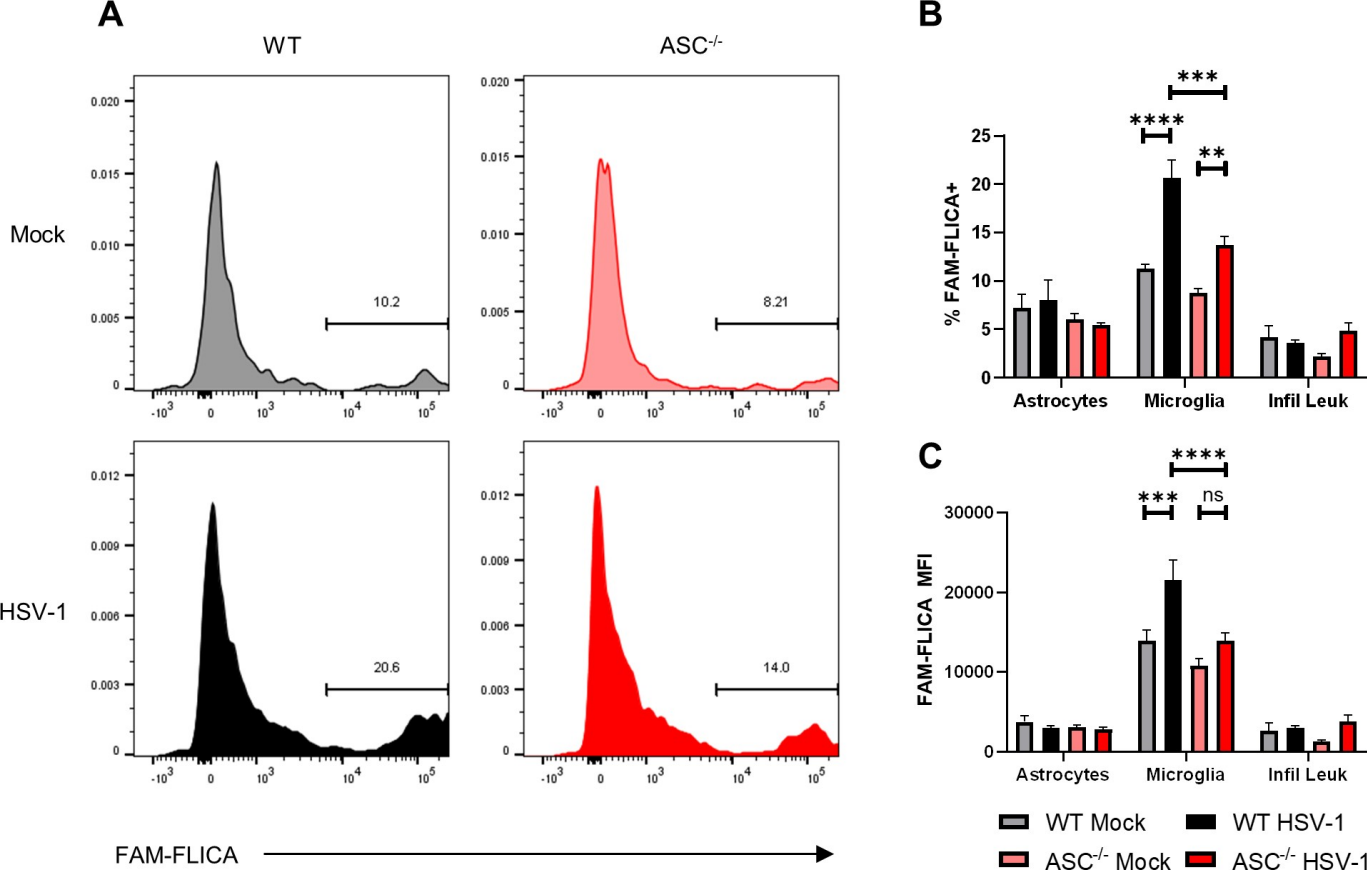
Microglial ASC-dependent inflammasomes are activated during HSE. Cell-specific caspase-1 activity was analyzed by flow cytometry and FLICA assay at day 3 post-infection following intracranial inoculation of 3 x 10^6^ PFU HSV-1 KOS. (A) Representative flow cytometry histograms of CD45^mid^CD11b+ microglia from mock-infected or HSV-1-infected WT and ASC^-/-^ mice stained with FLICA reagent to quantify active caspase-1. Percentages of each cell type that are FLICA+ (B) or FAM-FLICA mean fluorescence intensity (C) in astrocytes, microglia, and CD11b+ infiltrating leukocytes (n = 3–4 mice per group). After excluding multiplets and dead cells, cells were gated using the following strategy: astrocytes CD45^lo^/ACSA-2+, microglia CD45^mid^/CD11b+, infiltrating leukocytes CD45^hi^/CD11b+. Values are expressed as means ± SEM (**P < 0.01, ***P < 0.001, ****P < 0.0001). Results are representative of two independent experiments.

Microglia exhibited robust inflammasome activation during infection as evidenced by the representative flow cytometry histograms depicting FAM-FLICA intensity from WT and ASC^-/-^ mice either mock-infected or infected with HSV-1 (Fig 2A). The percentage of microglia that were FAM-FLICA^+^ increased significantly in both WT and ASC^-/-^ mice during infection, although the percentage of FAM-FLICA^+^ microglia was significantly lower in infected ASC^-/-^ mice compared to WT (Fig 2B). Because FAM-FLICA intensity is directly correlated with the quantity of activated caspase-1, we also measured the mean fluorescence intensity (MFI) of FAM-FLICA in each group of mice and saw a similar increase in microglia from infected WT mice. Of note, the MFI did not significantly increase in infected ASC^-/-^ microglia compared to mock infected cells (Fig 2C). Caspase-1 activity in ASC^-/-^ microglia was not completely abrogated, suggesting that ASC-independent non-canonical inflammasome pathways are also activated during infection. Surprisingly, caspase-1 activity in the infiltrating CD11b^+^ leukocyte populations from both WT and ASC^-/-^ mice did not increase during infection (Fig 2B). Astrocytes also had low levels of caspase-1 activity that did not increase with infection (Fig 2B). These data demonstrate that microglia are the key drivers of ASC-dependent inflammasome activity during HSE.

### IL-1β production is microglia-dependent in an organotypic brain slice culture model of HSV encephalitis

We demonstrated that microglia isolated from HSV-infected brains have increased caspase-1 activity (Fig 2). To validate the importance of microglia in inflammasome activation and IL-1β production during HSE we used an organotypic brain slice culture model of HSV-1 infection. Our group and others have previously used brain slice cultures (BSCs) to model HSV-1 encephalitis, and they are amenable to selective microglia depletion using clodronate liposomes [3,28].

**Fig 3:**
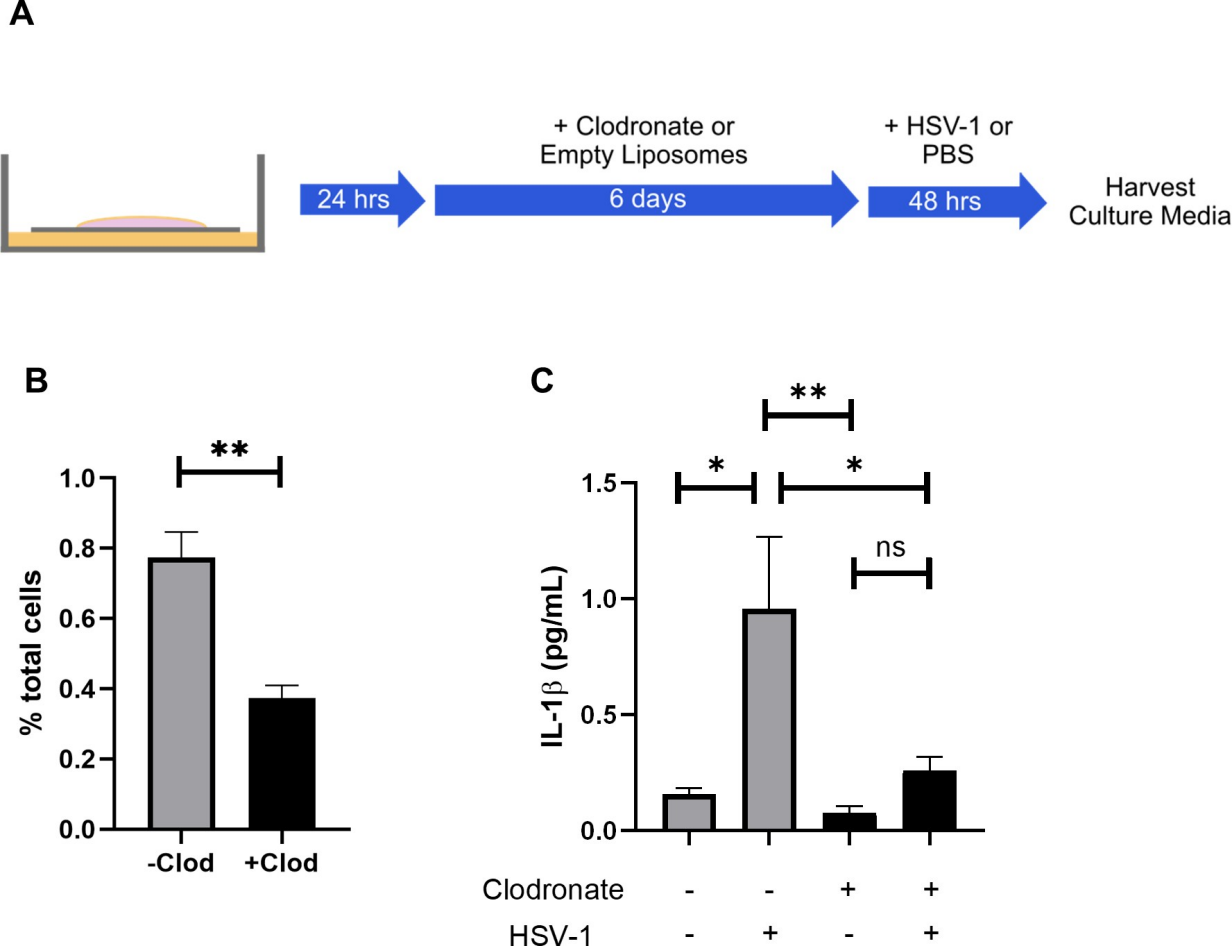
IL-1β production is microglia-dependent in an organotypic brain slice culture model of HSV encephalitis. Microglia were depleted from organotypic brain slice cultures by incubation with clodronate-filled liposomes and IL-1β production was quantified after HSV-1 infection. (A) Diagram of experimental set-up. Slices were allowed to settle for 24 hours in culture before incubation with either clodronate-filled or empty liposomes. Slices were then infected with 10^6^ PFU HSV-1 KOS and culture media was collected at 48 hour post-infection. (B) Microglia quantification by flow cytometry after 6 days of clodronate or empty liposome treatment (n = 4 slices per group). (C) Protein levels of IL-1β as quantified by ELISA (n = 3 slices per group). Values are expressed as means ± SEM (*P < 0.05, **P < 0.01).

Brain slices were obtained from WT mice and allowed to recover in culture for 24 hours before incubation with media containing either clodronate-containing liposomes or an equivalent concentration of empty liposomes as a control (Fig 3A). After six days of incubation with clodronate, depletion of microglia was confirmed by flow cytometry (Fig 3B). Slices were then either infected with HSV-1 or mock-infected with vehicle control and incubated for 48 hours, a time point previously determined to be associated with peak viral titer in brain slice cultures [3]. We collected culture media and quantified IL-1β by ELISA. Consistent with our prior results, IL-1β levels sharply rose after HSV-1 infection in BSCs without microglial depletion (Fig 3C). IL-1β did not increase during infection in microglia-depleted BSCs and was significantly lower at baseline than non-depleted BSCs, demonstrating that inflammasome activation and IL-1β release depend on microglia in a model of HSE (Fig 3C).

### Glial inflammasome activation drives expression of the monocyte chemokine CCL6 during HSV-1 infection

**Fig 4:**
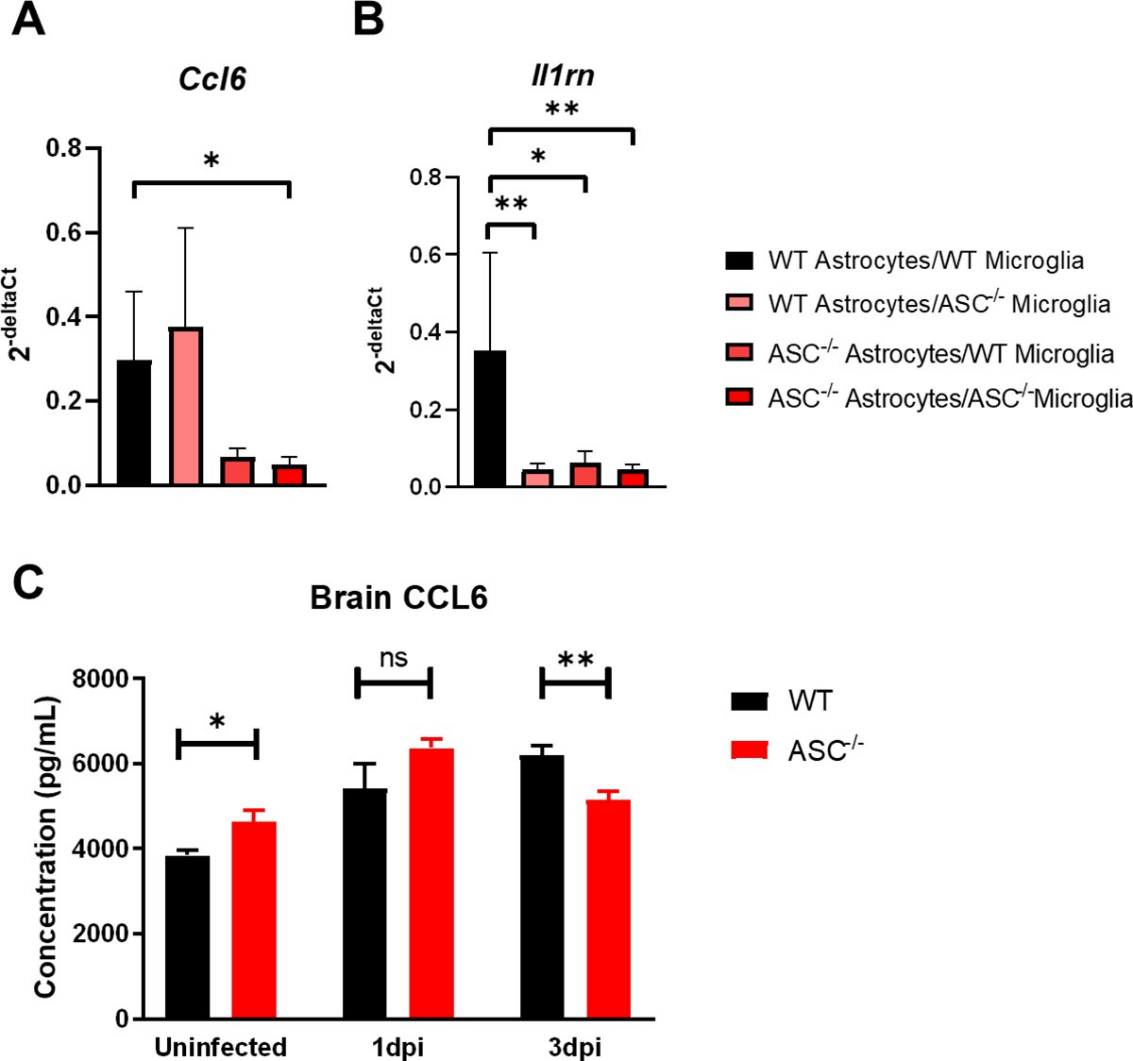
Glial inflammasome activation drives expression of the monocyte chemokine CCL6 during HSV-1 infection. Glial co-cultures were infected at an MOI of 5 and cellular RNA was isolated at 12 hours post-infection. Relative gene expression of (A) *Ccl6* and (B) *Il1rn* in glial co-cultures (n = 3–4 per group). (C) CCL6 protein levels from the brains of either uninfected or infected mice at days 1 and 3 post-infection (n = 4–5 per group). Values for gene expression are expressed as means ± SD and values for CCL6 levels are expressed as means ± SEM (*P < 0.05, **P < 0.01).

Our findings demonstrated that ASC-dependent inflammasomes increase mortality in HSE, and that microglia are the main contributors to inflammasome activity in the brain during HSV infection. To better understand the inflammasome-dependent immune signaling mechanisms in microglia that contribute to immunopathology in HSE, we screened for ASC-dependent pro-inflammatory cytokines and chemokines and validated their expression in vivo. Although microglia were identified to be the key inflammasome mediators in vivo (Figs 2 and 3), the innate immune response to HSV-1 has been shown to involve cross-talk between both microglia and astrocytes [29]. Therefore, we used a mixed glial cell culture model to screen for cytokines and chemokines that are induced by astrocytic or microglial inflammasome signaling. Microglia and astrocytes were isolated from WT and ASC^-/-^ mice and co-cultures were established with ASC^-/-^ astrocytes, ASC^-/-^ microglia, or both. After in vitro HSV-1 infection, gene expression was determined by quantitative real-time PCR array. Two genes, *Ccl6* and *Il1rn*, were significantly downregulated in at least one of the culture conditions containing ASC^-/-^ cells compared to cultures with only WT cells (S1 Fig). *Ccl6* gene expression in ASC^-/-^ astrocytes and ASC^-/-^ microglia cultures was significantly lower than in the WT astrocyte and microglia cultures (Fig 4A). Thus, both astrocytic and microglial ASC contribute to CCL6 expression in vitro. The chemokine CCL6 is a monocyte chemoattractant [30,31]. The gene *Il1rn* was also downregulated in all three culture conditions with ASC^-/-^ astrocytes and/or microglia (Fig 4B). *Il1rn* encodes the IL-1 receptor antagonist, IL-1RA, that acts as a negative regulator of IL-1β activity [32]. As a negative regulator, the expression of *Il1rn* is likely tightly linked to inflammasome activity, explaining its reduced expression in these cultures.

To validate the role of ASC in CCL6 expression in vivo, we measured CCL6 concentrations in the brains of either uninfected WT and ASC^-/-^ mice or at days 1 and 3 post-infection. Similar to IL-18 concentrations (Fig 1D), CCL6 was slightly higher in uninfected ASC^-/-^ mice compared to WT (Fig 4C). CCL6 was not significantly different between WT and ASC^-/-^ mice at day 1 post-infection, but by day 3 post-infection CCL6 levels were lower in ASC^-/-^ mice compared to WT mice (Fig 4C). Although early CCL6 expression does not depend on ASC, increased expression of this chemokine is ASC-dependent later in infection.

### ASC-dependent inflammasomes promote macrophage infiltration of the CNS during HSE

**Fig 5:**
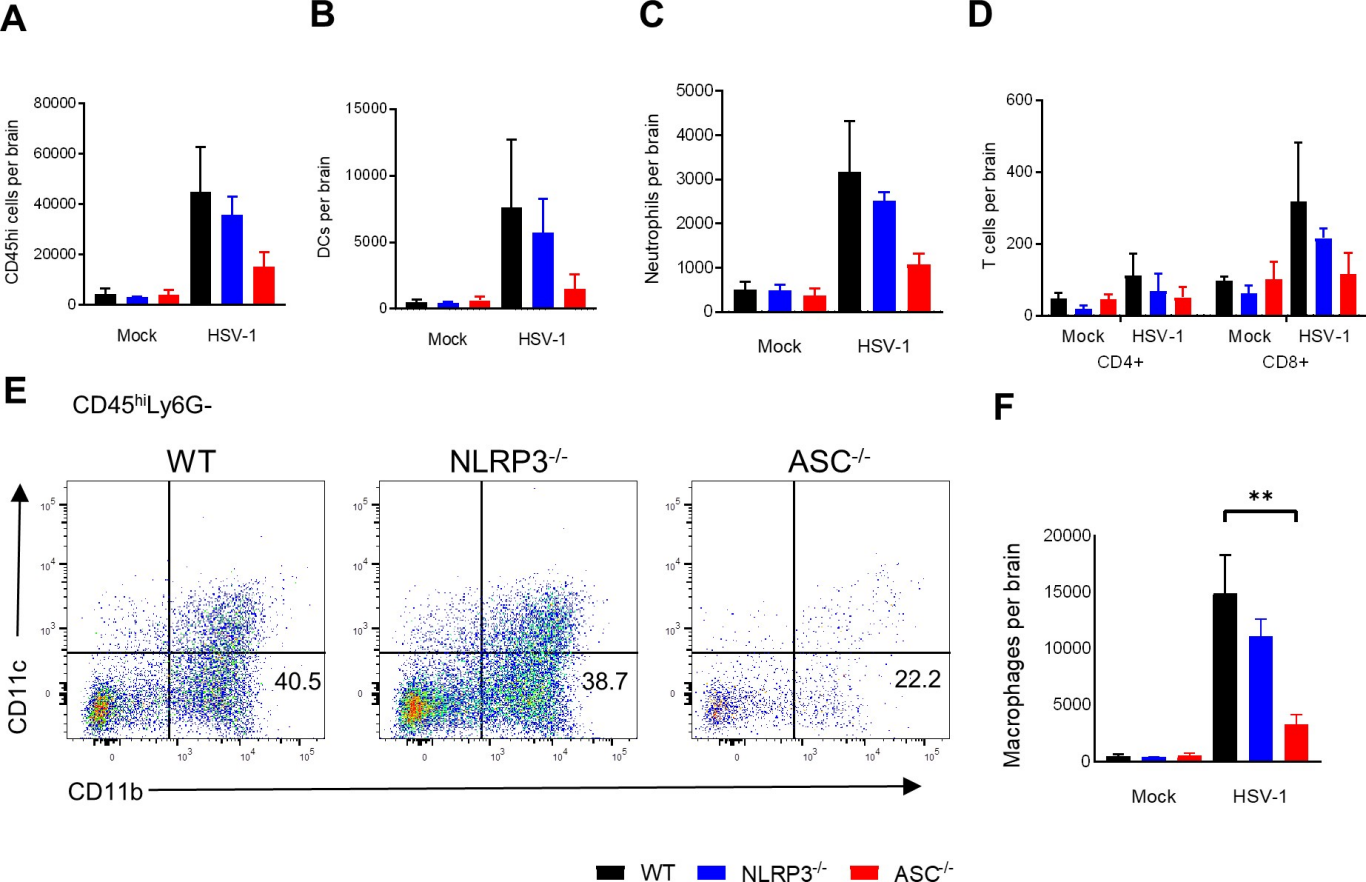
ASC-dependent inflammasomes promote leukocyte migration into the CNS during HSV-1 encephalitis. Infiltrating leukocytes in the brain were analyzed by flow cytometry in WT, NLRP3^-/-^, and ASC^-/-^ mice at day 3 post-infection after intracranial inoculation with 3 x 10^6^ PFU HSV-1 KOS. Total CD45^hi^ cells (A), dendritic cells (B), neutrophils (C), CD4+ and CD8+ T cells (D). Representative FACS plots of CD11c and CD11b expression within CD45^hi^ and Ly6G- cells. Macrophages are gated as CD11b+ and CD11c- (E). Absolute numbers of macrophages (F) calculated from numbers of total cells at day 3 post-infection (n = 5, 3 replicates for myeloid cells; n = 3–4, 2 replicates for T cells). After excluding multiplets, cell types were gated within the CD45^hi^ population using the following gating strategy: macrophages/monocytes Ly6G-/CD11c-/CD11b+, dendritic cells Ly6G-/CD11c+, neutrophils Ly6G+/CD11b+/CD11c-, T cells CD3+/CD4+ or CD3+/CD8+. No significant decrease was observed between WT and ASC^-/-^ mice for total CD45^hi^, DCs, neutrophils, or T cells. Values are expressed as means ± SEM (**P < 0.01).

Leukocyte infiltration into the brain has been shown to be important for controlling replication of HSV-1, yet the infiltration of CXCR3^+^ monocytes and T cells has been associated with detrimental inflammation in HSE [33,34]. We demonstrated that ASC^-/-^ mice have lower CCL6 concentrations at day 3 post-infection. Based on this finding, we hypothesized that ASC^-/-^ mice have reduced CNS infiltration of leukocytes at that time point. We examined several immune cell types including total infiltrating CD45^hi^ cell populations, macrophages, neutrophils, dendritic cells (DCs), and CD4^+^ and CD8^+^ T cells. Absolute numbers of total CD45^hi^ cells (Fig 5A), neutrophils, DCs, and both CD4+ and CD8+were reduced in ASC^-/-^ mice (Fig 5B, 5C and 5D) although the differences did not reach statistical significance. Notably, absolute numbers of infiltrating macrophages were significantly reduced in ASC^-/-^ compared to WT mice infected with HSV-1, suggesting that ASC-dependent inflammasomes drive the infiltration of this particular cell type (Fig 5E and 5F). Consistent with our survival and cytokine expression data, there was no difference in macrophage infiltration between WT and NLRP3^-/-^ mice (Fig 5F).

The significantly increased infiltration of macrophages in WT mice is consistent with the elevated CCL6 levels found at day 3 post-infection in Fig 4D. These results demonstrate that ASC is critical for the recruitment of macrophages into the CNS during HSE.

## Discussion

We report that the inflammasome adaptor protein, ASC, contributes to mortality in a murine model of HSE. By contrast, mice deficient in the inflammasome sensor components NLRP3 and AIM2 did not differ in survival from WT mice. We demonstrated that ASC-dependent inflammasomes contribute to mortality without affecting viral replication. ASC was also critical for the generation of the pro-inflammatory cytokines IL-1β and IL-18, suggesting that ASC-dependent inflammasomes are the primary contributors to the release of these cytokines during HSE. We identified microglia as the primary mediators of the inflammasome response in vivo and in an organotypic brain slice culture model of HSV-1 infection. Using both in vitro infections of glial cultures and our in vivo HSE model, we found that expression of the macrophage chemoattractant CCL6 depends on ASC-dependent inflammasomes. Finally, reduced CCL6 in ASC^-/-^ mice was associated with decreased infiltration of macrophages to the CNS during infection.

ASC is the key adaptor for canonical inflammasomes, acting as a bridge between the sensor and procaspase-1 through its PYD (pyrin domain) and CARD (caspase activation and recruitment domain) domains, respectively [35]. HSV-1 infection in vitro has been shown to activate several of these inflammasomes that act through ASC, including NLRP3, AIM2, and IFI16 [18,19,21], but the extent to which these contribute to disease in the CNS were unknown. The role of inflammasomes in HSV-1 pathogenesis in vivo has been largely limited to murine models of corneal HSV-1 infection. Prior studies found that corneal infection of NLRP3^-/-^ mice results in more severe immunopathogenesis [23], and that corneal HSV-1 infection induces the NLRP3, NLRP12, and IFI16 inflammasomes [36]. A previous study found that IL-1β-deficient mice were more susceptible in an intranasal model of HSV-1 infection [25]. This discrepancy is likely due to the route of infection, with IL-1β being important for preventing neuroinvasion or restricting peripheral replication in the nasal mucosa but detrimental once the virus has gained access to the CNS. In an intracranial model of HSE, our study suggests that multiple ASC-dependent inflammasomes contribute to pathogenesis by driving excessive neuroinflammation in the CNS, but that neither NLRP3 or AIM2 alone account for differences in survival or cytokine production. Interestingly, NLRP3 deficiency resulted in higher levels of IL-1β in the brain later in infection, suggesting a regulatory role for NLRP3 in modifying caspase-1 activity rather than simply increasing IL-1β activation in response to an immune stimulus. Our study suggests a complex immunomodulatory role for ASC in regulating multiple inflammasomes in the brain, without a dominant PRR component.

Importantly, our study identified microglia as key mediators of the inflammasome response in HSE, adding to mounting data that microglia contribute to pathology in HSV-1 CNS infection [37]. We also demonstrated downregulation of the chemokine CCL6 in cultures of ASC^-/-^ glia and in the brains of ASC^-/-^ mice at day 3 post-infection. Interestingly, CCL6 was elevated in both WT and ASC^-/-^ mice at day 1 post-infection. Taken together, these results suggest that after initial activation early in infection, ASC-dependent inflammasomes significantly influence immune cell infiltration later in infection by regulating CCL6 production and contribute to a prolonged neuroinflammatory response. CCL6 has been described as a monocyte chemoattractant through its putative receptor, CC-type chemokine receptor 1 (CCR1) [30,31]. Few studies have examined CCL6 in the brain, but it has been reported to be expressed in rat microglia and not astrocytes [38]. In a murine model of Venezuelan equine encephalitis virus, more virulent strains of the virus induce greater expression of CCL6 in the brain and subsequent infiltration of mononuclear cells into the parenchyma [39]. These studies are consistent with our finding of reduced macrophage infiltration in the brain in ASC^-/-^ mice, coinciding with reduced levels of CCL6, and may represent a common mechanism of adaptive immune cell recruitment to the brain following neurotropic virus infection. Together, our findings suggest that microglial inflammasome activation may serve as a key link between the early innate immune response to HSV-1 and prolonged neuroinflammation through the recruitment of infiltrating leukocytes.

Although components of the innate immune system are critical for controlling HSV-1 replication in the brain [3,4], our study found that ASC-dependent inflammasomes do not contribute to controlling viral replication. Improved survival in ASC^-/-^ mice in the setting of decreased inflammatory markers, with no influence on viral replication, indicates that inflammasomes are immunopathogenic in HSE. These results are consistent with studies demonstrating a key role for inflammasomes in driving neuroinflammation in response to other viral CNS infections [24,40,41]. In HSV-1 infection, neuronal damage can be caused by both viral replication and pathologic inflammation, which can also ultimately promote excitotoxicity, pyroptosis, and necroptosis [5,42]. As a result, the host response to infection represents a careful balance between an appropriate immune response and prolonged neuroinflammation. Immunopathology is clinically apparent through the standard use of acyclovir, an anti-viral therapeutic targeted to control HSV replication, which has significantly reduced mortality but not neurologic morbidity following HSV encephalitis [43]. This suggests that the host immune response significantly impacts neurologic outcomes in HSE beyond the direct detrimental effects of viral replication. Our data suggest that targeting inflammasome activation in HSE may shift the balance to a less detrimental immune response and improve outcomes after HSV infection.

Current antiviral therapy for HSV-1 is targeted to viral replication, but adjunctive corticosteroid treatment has been suggested to counteract immunopathology [44]. However, rational targeting of immune pathways offers the possibility of preventing harmful inflammation without impairing viral clearance, offering a synergistic approach to the treatment of a diverse spectrum of viral illnesses. Targeting inflammasome components has been explored in both neuroinflammatory disorders and pre-clinical models of viral disease. For example, the IL-1 receptor antagonist, anakinra, has been shown to be effective in improving outcomes in mouse models of Zika virus-induced placental dysfunction and Chikungunya virus arthritis [45,46]. Anakinra has also been used to treat patients with Behcet’s disease, an inflammatory disorder with CNS involvement [47], and has been demonstrated to cross the blood-brain barrier in humans [48]. This drug, and others that similarly target IL-1 signaling, may represent a better strategy to selectively target harmful inflammation during HSV-1 infection. Our study suggests that targeting the inflammasome response could prevent harmful inflammation with the potential to improve survival independent of viral clearance in HSE.

## Materials and methods

### Ethics statement

This study was carried out in adherence to the recommendations in the Guide for the Care and Use of Laboratory Animals of the National Institutes of Health. The protocol was approved by the Institutional Animal Care and Use Committee (IACUC) of Northwestern University (protocol IS00003204). Anesthesia for intracranial infections was performed using isoflurane. Euthanasia was performed using CO2 and cervical dislocation.

### Cells and virus

HSV-1 KOS was propagated in Vero cells and stored in -80°C until use. Plaque assays were used to determine viral titers as described previously [49]. Primary astrocytes and microglia were isolated from both male and female 6–8 week-old WT or ASC^-/-^ C57BL/6J mice by first generating single-cell suspensions using the Neural Tissue Dissociation Kit (P) (Miltenyi Biotec, San Diego, CA) following the manufacturer’s manual dissociation protocol. Astrocytes and microglia were then sequentially isolated using the anti-ACSA-2 and anti-CD11b microbead kits (Miltenyi Biotec) according to the manufacturer’s protocol. Primary cells were maintained in complete Dulbecco’s modified Eagle medium (DMEM + 10% FBS + 1% L-glutamate + penicillin/streptomycin) for one week prior to infection.

### HSV-1 Encephalitis model

Viral stocks were diluted to the appropriate concentration in phosphate-buffered saline containing 1% heat-inactivated serum and 0.1% glucose (PBS-GCS). To control for possible immune stimulation by cell debris in the viral inoculum, stocks for mock infections were prepared by diluting supernatant from mock-infected Vero cells to the same dilution factor as viral stocks with PBS-GCS. Wild-type C57BL/6J mice were obtained from the Jackson Laboratories (#000664). C57BL/6J NLRP3^-/-^ and ASC^-/-^ mice were obtained from Karen Ridge (Northwestern University) and Christian Stehlik (Northwestern University), respectively, and have been described previously [50,51]. C57BL/6J AIM2^-/-^ mice were obtained from the Jackson Laboratories (#013144). Mice were anesthetized with isoflurane prior to infection.

Viral inoculum or diluted Vero supernatant were delivered in 10 μL using a positive displacement syringe and 26-gauge needle with a needle guard. The needle was placed through the right parietal bone, lateral to the sagittal suture and equidistant to the lambda and bregma. For survival experiments, mice were infected with 3x10^4^ PFU of HSV-1 KOS and weighed daily over the course of the 14-day experimental period. Mice were sacrificed if they lost 30% of their starting weight. For time course tissue titering experiments, mice were infected as described above and sacrificed at the appropriate time point. Brains were collected for viral titering at mortality or the end of the experiment and homogenized in 1 mL DMEM containing penicillin/streptomycin and sonicated.

### ELISAs

For brain tissue ELISAs, mice were inoculated with 3 x 10^4^ PFU HSV-1 KOS and sacrificed at the appropriate time point. Samples were whole-brain homogenates. IL-1β (BMS6002), IL-18 (BMS618-3), and CCL6 (EMCCL6) ELISA kits (Thermo Fisher) were used according to the manufacturer’s instructions.

### Flow cytometry

Mice were inoculated with 3 x 10^4^ PFU HSV-1 KOS or mock-infected and sacrificed by transcardial perfusion with PBS at day 3 post-infection. Brains were dissociated using the Neural Tissue Dissocation Kit (P) (Miltenyi Biotec). For enumeration of infiltrating leukocytes, cells were first treated with TruStain FcX anti-CD16/32 antibody (Fc block-BioLegend; 93) before staining with the following antibodies: Ly6G APC-Cy7 (BioLegend; RB6-8C5), CD11c APC (BioLegend; N418), and CD11b BV421 (BD; M1/70). Flow cytometry was performed on a FACS CantoII (BD Biosciences, San Jose, CA, USA) and analyzed using FlowJo 10.1 software (Ashland, OR, USA).

For fluorescently-labeled inhibitor of caspase activity (FLICA) assays, cell suspensions were first incubated with a 1:1000 dilution of Live/Dead Fixable Aqua Dead Cell Stain Kit (Thermo Fisher Scientific) before incubation with the FAM-FLICA reagent according to the manufacturer’s protocol (ImmunoChemistry Technologies). Cells were treated with an Fc-blocking reagent (Fc block-BioLegend; 93) and stained with the following antibodies before being analyzed: ACSA-2 PE (Miltenyi Biotec; REA969), CD45 PerCP-Cy 5.5 (BD Biosciences; 30-F11), and CD11b PE-Cy7 (eBioscience; M1/70). Gating of positive populations was based on Fluorescence minus one control (FMO) samples.

### Glial Co-culture infections

1.5 x 10^5^ cells were infected at an MOI of 5 with HSV-1 KOS diluted in 0.3 mL PBS-GCS. PBS-GCS alone was used for mock infections. Cells were incubated with the inoculum and gently rocked at 37°C for 2 hours. The inoculum was then aspirated and cells were washed twice with PBS before replacing the growth media. Cellular RNA was isolated 12 hours later using TRIzol reagent (Thermo Fisher Scientific) according to the manufacturer’s protocol and quantified using a NanoDrop 1000.

### RT-PCR Cytokine and Chemokine array

Cellular RNA from glial cell infections was reverse transcribed using the RT^2^ First Strand Kit (QIAGEN) with 0.4 μg RNA per sample. The relative expression of 84 pro-inflammatory cytokines and chemokines was then quantified using the RT^2^ Profiler PCR Array PAMM-011Z (QIAGEN). Gene expression was analyzed with the RT^2^ Profiler PCR Array analysis software from the GeneGlobe Data Analysis Center using the ΔΔCt method with a Ct cutoff of 35. Expression was normalized to the housekeeping gene *Gapdh*. Genes for which fewer than three samples per group had detectable levels of transcript were excluded from analysis. P values were calculated based on a two-tailed Student’s t-test of 2^-ΔCt^ values for each gene and p<0.5 was considered significant. Relative gene expression was visualized using the freely available online tool Heatmapper [52].

### Organotypic Brain slice cultures

Brain slice cultures were prepared from P6-10 neonatal mice as described previously [3]. Membranes with brain slices were placed in six-well plates with 1 mL of filter culture media [50% (vol/vol) minimal essential media, 25% (vol/vol) HBSS, 25% (vol/vol) heat-inactivated horse serum, 0.044% NaHCO_3_, 2 mM glutamine, 10 U/mL penicillin]. Slices were cultured for 24 hours before being transferred to filter culture media containing either 25 mg/mL clodronate-containing liposomes or an equivalent concentration of empty liposomes (Encapsula NanoSciences). Slices were then cultured for six days in clodronate-containing media or media with empty liposomes with the media changed every two days. After six days in liposome-containing culture, slices were infected with 10^6^ PFU of HSV-1 KOS in 1 mL of PBS-GCS. After two hours, the viral inoculum was aspirated and membranes were transferred to fresh filter culture media. Media was collected for ELISA analysis at 48 hours post-infection.

### Statistics

All statistics were calculated using GraphPad Prism 7.0 software. For survival experiments, logrank analysis with the Bonferroni adjustment for multiple comparisons was performed. Viral titers and cytokines were analyzed using multiple t-tests with the Holm-Sidak correction for multiple comparisons. FLICA results were analyzed using two-way ANOVA with Tukey’s multiple comparison test. Gene expression array results were analyzed using the delta-delta Ct method with significant results determined by two-tailed t-tests of 2^-ΔCt^ values for each gene. Cell enumeration by flow cytometry was analyzed using one-way ANOVA with Dunnett’s multiple comparison test.

## Supporting information

S1 Fig Normalized relative gene expression in glial co-cultures. Heat map of z-score normalized relative gene expression of pro-inflammatory cytokines and chemokines for each culture condition. *Ccl6* and *Il1rn* are highlighted as having significant differences between groups.
(TIF)
