# Supplementary figures and images for: ASC-dependent inflammasomes contribute to immunopathology and mortality in herpes simplex encephalitis
Source: PLoS Pathog. 2021 Feb 1;17(2):e1009285. doi: 10.1371/journal.ppat.1009285 (PMC7877773; doi:10.1371/journal.ppat.1009285)

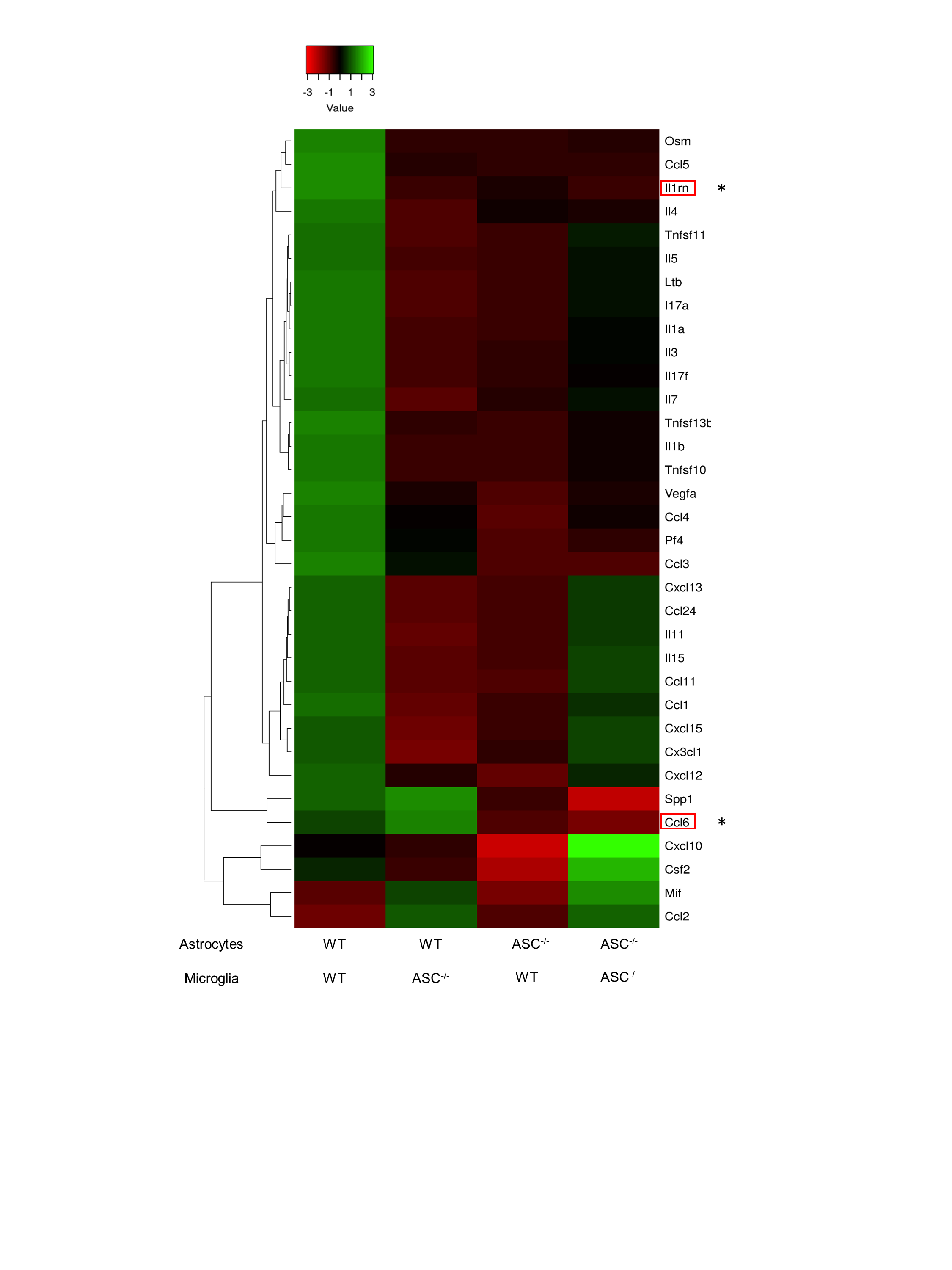

Supplement: S1 Fig — Heat map of z-score normalized relative gene expression of pro-inflammatory cytokines and chemokines for each culture condition. Ccl6 and Il1rn are highlighted as having significant differences between groups. (TIF) [file ppat.1009285.s001.tif]
